# Which sets of elementary flux modes form thermodynamically feasible flux distributions?

**DOI:** 10.1111/febs.13702

**Published:** 2016-03-31

**Authors:** Matthias P. Gerstl, Christian Jungreuthmayer, Stefan Müller, Jürgen Zanghellini

**Affiliations:** ^1^ Department of BiotechnologyUniversity of Natural Resources and Life SciencesViennaAustria; ^2^ Austrian Centre of Industrial BiotechnologyViennaAustria; ^3^ Johann Radon Institute for Computational and Applied MathematicsAustrian Academy of SciencesLinzAustria

**Keywords:** constraint‐based analysis, metabolic pathway analysis, metabolomics, steady‐state flux cone, thermodynamics

## Abstract

Elementary flux modes (EFMs) are non‐decomposable steady‐state fluxes through metabolic networks. Every possible flux through a network can be described as a superposition of EFMs. The definition of EFMs is based on the stoichiometry of the network, and it has been shown previously that not all EFMs are thermodynamically feasible. These infeasible EFMs cannot contribute to a biologically meaningful flux distribution. In this work, we show that a set of thermodynamically feasible EFMs need not be thermodynamically consistent. We use first principles of thermodynamics to define the feasibility of a flux distribution and present a method to compute the largest thermodynamically consistent sets (LTCSs) of EFMs. An LTCS contains the maximum number of EFMs that can be combined to form a thermodynamically feasible flux distribution. As a case study we analyze all LTCSs found in *Escherichia coli* when grown on glucose and show that only one LTCS shows the required phenotypical properties. Using our method, we find that in our *E. coli* model < 10% of all EFMs are thermodynamically relevant.

**Abbreviations**
ATP: adenosine triphosphate
EFM: elementary flux mode
EFMA: EFM analysis
LTCS: largest thermodynamically consistent set
MILP: mixed integer linear program
tEFMA: thermodynamic EFMA
TF: thermodynamically feasible

## Introduction

Elementary flux mode analysis (EFMA) is a key concept in constraint‐based modeling, which allows a metabolic network to be decomposed into irreducible functional building blocks, called elementary flux modes (EFMs). An EFM corresponds to a minimal set of reactions that can operate at steady state, thereby using all irreversible reactions in the appropriate direction 1. Here minimal means that no reaction can be removed from the set without losing the ability to form a non‐zero steady‐state flux. EFMs represent functional units in a metabolic network. In fact, every steady‐state flux can be represented as a nonnegative linear combination of EFMs *without cancelations* [S. Müller and G. Regensburger, unpublished results;^2^, ^3^]; it is always possible to find such a ‘conformal sum’ 4, 5. The no‐cancelation rule guarantees that every reaction proceeds in the same direction in all contributing EFMs and accounts for the fact that, for given metabolite concentrations, only one direction of a reversible reaction is thermodynamically feasible (TF).

From a systems‐biology point of view, the advantage of an unbiased decomposition of a metabolic network into EFMs lies in the ability to fully characterize the metabolic capabilities of an organism. This can be used, for instance, to analyze cellular robustness 6 or in metabolic engineering to turn wild‐type organisms into so‐called ‘networks of minimal functionality’ 7. These (mutant) networks are typically made up of very few, desired EFMs while all the unwanted (wild‐type) functionality is eliminated by appropriately selected gene knockouts 8, 9, 10, 11, 12. Therefore, EFMA is an ideal tool for metabolic engineering and synthetic biology to rationally design optimal cell factories 13.

However, a complete EFMA is currently limited to medium‐scale metabolic models since the number of EFMs explodes with the size of the metabolic network 14. Using a massively parallelized approach, the largest complete EFMA reported to date found almost two billion EFMs in a metabolic reconstruction of *Phaeodactylum tricornutum* with 318 reactions 15. Alternative methods do not aim to find all EFMs, but limit their scope to find subsets. Subsets are selected randomly 16, 17 or based on support information 18 or subject to additional constraints 19, 20, 21. The question remains, however, whether or not these subsets are biologically relevant. To find relevant EFMs, Rezola *et al*. 22 used gene expression data and identified small subsets of EFMs that successfully characterized and described key metabolic features in different tissues. Similarly, Jol *et al*. 23 and our group 24, 25 used experimentally determined metabolomes to identify all EFMs that are TF. However, even if two EFMs are TF, it does not necessarily mean that their convex superposition is TF as well 23. This raises the question which TF EFMs can be combined in a convex superposition such that the resulting flux distribution will again be TF.

Here, we expand on our earlier work on the thermodynamics of EFMs 24, 25 and present a mixed integer linear program (MILP) that identifies the largest thermodynamically consistent sets (LTCSs) of EFMs. These LTCSs are characterized by the fact that every nonnegative linear combination of its EFMs results in a TF flux distribution. Moreover, we show that physico‐chemical constraints alone already severely limit the metabolic capabilities of an organism since only a small fraction of all EFMs are required to represent TF flux distributions. This confirms the hypothesis that, under given conditions, only a few EFMs are actually biologically relevant and accessible to an organism 26.

## Results

### Theory

#### Notation and model assumptions

We consider a metabolic core model of *Escherichia coli* 25, referred to as M‐glc, to study growth on minimal medium (containing ammonia, oxygen, phosphate, protons and water) with glucose as the sole carbon source. The model is characterized by its stoichiometric matrix $S\in R^{m\;\times \;r}$ with *m* = 76 internal metabolites and *r* = 101 reactions of which 48 are reversible. The network contains *n* ^tot^ = 169 916 EFMs of which *n* = 32 374 EFMs are TF. Reactions and intracellular metabolites are thermodynamically characterized by their Gibbs free energy of reaction Δ_r_ *G* and their standard transformed Gibbs energy of formation Δ_f_ *G*′^0^, respectively. The latter is estimated using a pH of 7 and an ionic strength *I* = 0.15 m at a temperature *T* = 310.15 K (37°C), according to Alberty 27.

#### Thermodynamic EFMA

Thermodynamic constraints are often utilized to augment classical constraint‐based approaches 28. For instance, Hoppe *et al*. 29 developed a metabolomics‐integrated flux‐balance analysis. Similarly, we developed thermodynamic EFMA (tEFMA) 25, a computational tool that calculates all TF EFMs in a metabolic reconstruction. tEFMA exploits the fact that, according to the second law of thermodynamics, an EFM *e* ^*i*^ is TF if and only if all reactions *j* which support *e* ^*i*^ proceed in the direction of negative Gibbs free energy 30, that is, Δ_r_ *G*_*j*_ < 0 for all reactions *j* with $e_{j}^{i}\;>\;0$. Based on this fundamental property, tEFMA avoids the calculation of thermodynamically infeasible EFMs, which drastically reduces the computational burden of an EFMA and makes the analysis of large scale metabolic networks feasible without losing any biologically relevant information 24. Thus, given an experimentally measured cellular metabolome, the standard Gibbs free energy of formation for as many cellular metabolites as possible, and a metabolic reconstruction, tEFMA returns the complete set of TF EFMs consistent with the measurements.

#### Largest thermodynamically consistent sets

**Figure 1:**
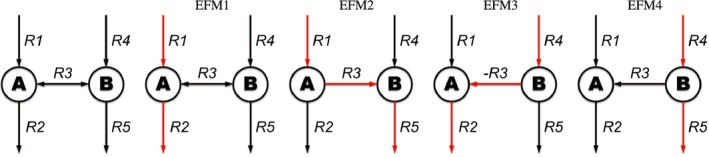
Simple example network (leftmost panel), containing two internal metabolites (A and B) and five reactions (of which only *R*3 is reversible), and its four EFMs (successive panels to the right; EFMs highlighted in red).

tEFMA computes all *n* TF EFMs of a network. However, not every set of TF EFMs is necessarily thermodynamically consistent. For example, two EFMs that utilize the same reversible reaction in different directions cannot be active simultaneously. This is illustrated in the simple example network in Fig. 1. Suppose that all four EFMs are TF. Yet, EFM2 and EFM3 cannot be active at the same time since Δ_r_ *G* cannot be smaller than zero for both directions of the reversible reaction R3. In other words, thermodynamics implies the no‐cancelation rule mentioned in the introduction. In this example, the sets {EFM1, EFM2, EFM4} and {EFM1, EFM3, EFM4} are the LTCSs, and its elements can contribute to a TF steady‐state flux.

**Figure 2:**
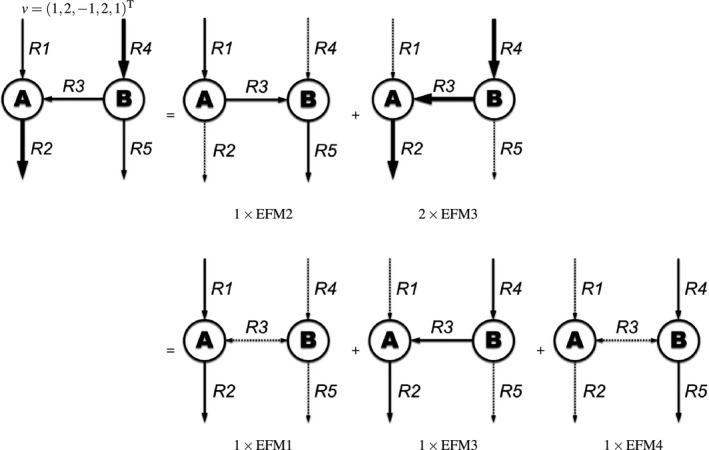
Exemplary TF overall flux distribution (top left panel) in the toy network of Fig. 1, a thermodynamically inconsistent EFM decomposition (top centre and top right panels), and a thermodynamically consistent EFM decomposition (bottom panels). Non‐zero flux values are represented by an appropriately scaled line thickness of the reaction arrows. Zero flux values are represented by dotted reaction arrows. A flux from A to B across the reversible reaction *R*3 is counted positive; a backward flux is counted negative. The uptake fluxes of all EFMs are normalized to 1.

For instance, consider the TF steady‐state flux *v* ^T^ = (*v*_1_,…,*v*_5_) = (1, 2, −1, 2, 1). Obviously, the flux can be decomposed as *v* = EFM2 + 2 × EFM3 (see Fig. 2 for an illustration). Although EFM2 and EFM3 are TF individually, they cannot be active simultaneously (no‐cancelation rule). Hence, this decomposition is not thermodynamically consistent. In contrast, the representation *v* = EFM1 + EFM3 + EFM4 is thermodynamically consistent. Indeed, these three EFMs form one of the LTCSs above, and every TF steady‐state flux can be represented by elements of one LTCS. The example raises the question of how all LTCSs can be computed systematically, given a set of TF EFMs.

*Definition: LTCS*. A set of TF EFMs is called *thermodynamically consistent* if every nonnegative linear combination of its elements is TF. Moreover, a set of TF EFMs is called an *LTCS*.

- if the set is thermodynamically consistent, and
- if no other TF EFM can be added to the set without losing thermodynamic consistency.

We first determine an LTCS ℒ_1_ of maximal cardinality. Alternative LTCSs of maximal cardinality or LTCSs of lower cardinality, ℒ_1_ with *l* > 1, can be found by successively excluding already existing LTCSs; see constraint (2) below

An LTCS ℒ_1_ of maximal cardinality is an optimal solution to the MILP

(1a) $$\max_{\lambda^{1},\ln c}\;\sum_{i=1}^{n}\lambda_{i}^{1},\;\text{where}\;\lambda^{1}\in {\left\{0,1\right\}}^{n},\ln c\in R^{m},$$

(1b) $$\text{s.t.}\;{\Delta_{r}G}_{j}<0\;\text{if}\;\rho_{j}\geq 1,\;\text{for all}\;j\in \left\{1,\ldots ,r\right\},$$

where

(1c) $$\rho_{j}=\sum_{i=1}^{n}\lambda_{i}^{1}\delta_{ij},$$

(1d) $$\delta_{ij}=\left\{\begin{array}{cc} 1 & \mathrm{if}\;e_{j}^{i}>0,\\ 0 & \mathrm{otherwise} \end{array}\right.$$

and

(1e) $${\Delta_{r}G}_{j}=\sum_{k=1}^{m}{\Delta_{f}G}_{k}^{\prime }S_{kj},$$

(1f) $$\Delta_{f}G_{k}^{\prime }={\Delta_{f}G}_{k}^{\prime 0}+RT\ln\left(c_{k}/c_{0}\right),\;c_{0}=1M,$$

(1g) $$\ln\left(c_{k}^{\min}/c_{0}\right)\leq \ln\left(c_{k}/c_{0}\right)\leq \ln\left(c_{k}^{\max}/c_{0}\right).$$

We use the superscript in λ^1^ to denote its association with the LTCS ℒ_1_. Briefly, λ^1^ indicates the presence ($\lambda_{i}^{1}=1$) or absence ($\lambda_{i}^{1}=0$) of EFM *e* ^*i*^ in the LTCS ℒ_1_, and we maximize $\sum_{i\;=\;1}^{n}\lambda_{i}^{1}$, that is, the cardinality of ℒ_1_, by varying the contributing EFMs and the (logarithms of the) metabolite concentrations *c*_*k*_, as stated in Eqn (1a).

Most importantly, δ_*ij*_ indicates if EFM *e* ^*i*^ is supported by reaction *j*, and ρ_*j*_ counts the number of EFMs supported by reaction *j*, as stated in Eqns (1d) and (1c). If at least one EFM is supported by reaction *j*, that is, ρ_*j*_ ≥ 1, then this reaction must be TF, according to the main constraint (Eqn 1b). Equivalently, if reaction *j* is infeasible, then ρ_*j*_ = 0 and hence $\lambda_{i}^{1}$ is forced to 0 for all EFMs *i* supported by reaction *j*.

Finally, Eqns (1e) and (1f) determine the Gibbs free energy of reaction *j*, given the (logarithms of the) metabolite concentrations. Thereby, *S*_*kj*_ denotes the elements of the stoichiometry matrix. The inequalities (Eqn 1g) constrain the metabolite concentrations.

Alternative optima and suboptimal solutions λ^*l*^ with *l* > 1 (and the corresponding LTCS ℒ_1_) can be found by successively excluding already existing solutions λ^*j*^ with *j* ∈ {1,…,*l*−1} from the MILP 9. This is achieved by successively adding the constraint

(2) $$\sum_{i\in Z}\lambda_{i}^{l}\geq 1,\;Z=\left\{i|\lambda_{i}^{l}=0\;\;\text{for all}\;\;j\in \left\{1,\ldots ,l-1\right\}\right\}$$

The process terminates when the MILP becomes infeasible, and no further solutions are found.

In the following, we computed all LTCSs for *E. coli* grown on minimal medium with glucose as the sole carbon source. Subsequently, we narrowed down the number of LTCSs to one by successively considering additional yield, expression and flux data.

### LTCSs are much smaller than the set of TF EFMs

**Figure 3:**
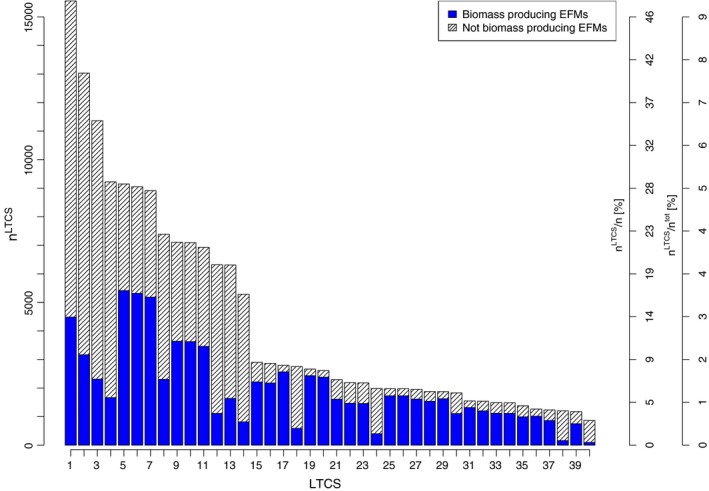
Cardinality of the LTCSs $L_{i}$ as a function of the set index *i*. Absolute numbers of EFMs *n* ^LTCS^ are displayed on the left axis and additionally displayed as percentages of the *n* ^tot^ EFMs and the *n* TF EFMs on the right axis.

As a matter of fact, there are 40 LTCSs for *E. coli* grown on minimal medium with glucose as the sole carbon source. The largest LTCS contains 15 560 EFMs. This corresponds to only 9% of all EFMs or 47% of the TF EFMs (see Fig. 3).

Moreover, we found that in general the relative frequency of biomass producing EFMs is larger in LTCSs of smaller cardinality. Still, the average number of biomass producing EFMs per LTCS (1984 ± 69%) appeared to be more stable than the average number of EFMs per LTCS (4316 ± 86%).

### Yield data identifies biologically relevant LTCSs

An LTCS represents the metabolic capabilities of *E. coli*, under the conditions specified in the model. To characterize these capabilities, we used maximum yield parameters $Y_{x_{i}/\text{glc}}$. $Y_{x_{i}/\text{glc}}$ was defined as the maximum of the yields of all EFMs in an LTCS for a specific product *x*_*i*_. To identify the biologically relevant LTCSs, we used typical growth parameters obtained by Andersen and Meyenburg 31.

**Figure 4:**
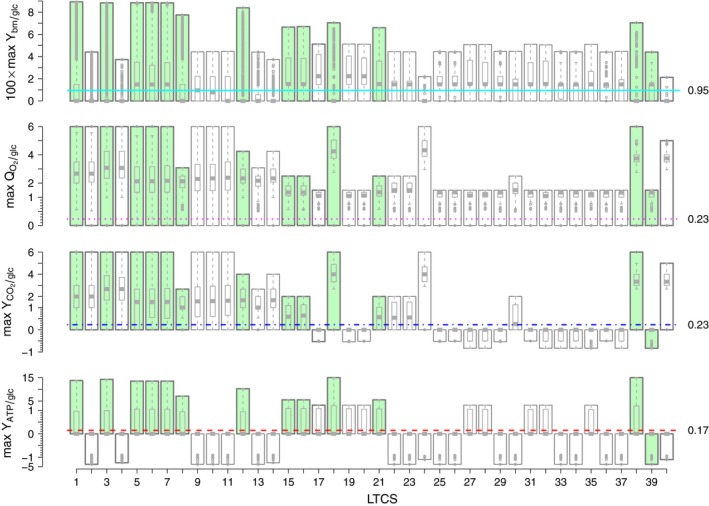
Different maximum yields (bars) for growth on glucose (glc) as functions of the LTCS index and in comparison with experimentally determined yields (horizontal lines) as measured by Andersen and Meyenburg 31. The measured yields are printed next to the lines on the right hand side. Colored bars indicate those LTCSs whose maximal yields are concurrently larger than the measured yields in all four cases. The overlaying boxplots indicate the yield distributions of the EFMs within an LTCS. ATP, adenosine triphosphate; bm, biomass; O_2_, dioxygen; CO_2_, carbon dioxide.

**Figure 5:**
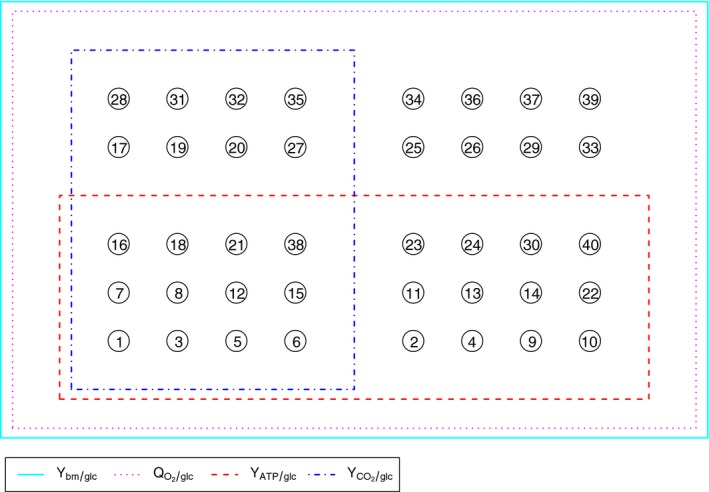
Venn diagram of the barplot in Fig. 4. LTCSs are grouped based on their reachable yields.

Figure 4 shows different maximal yields for each LTCS in comparison with measured data. Note that those maximal yields were only achieved by a few EFMs within an LTCS. Most of the EFMs in any given LTCS had a smaller or even zero yield. Thus, every yield between the maximum and zero can be achieved by a suitable combination of a maximum yield EFM and a zero yield EFM. In particular, if the measured yield is below the maximum, then it can be achieved by an appropriate combination of EFMs. Conversely, if the achievable maximum yield of an LTCS is below the measured value, then no combination of EFMs can result in the observed yield, and those LTCSs can be excluded from further analysis. We found that only 12 out of 40 LTCSs were consistent with the measured yields (see Fig. 5). These 12 sets can be calculated directly if the measured yields are used as additional constraints in the MILP (Eqn (1a), (1b), (1c), (1d), (1e), (1f), (1g)). Then the modified MILP reads

(3a) $$\max_{\lambda^{\left(1\right)},\ln c}\sum_{i=1}^{n}\lambda_{i}^{\left(1\right)},\;\lambda^{\left(1\right)}\in {\left\{0,1\right\}}^{n},\ln c\in R^{m},$$

(3b) $$\text{s.t.}\;{\Delta_{r}G}_{j}<0\;\text{if}\;\rho_{j}\geq 1,\;\text{for all}\;j\in \left\{1,\ldots ,r\right\},$$

(3c) $$\sigma_{u}\geq 1,\text{for all}\;u\in \left\{\text{ATP},\;{\mathrm{CO}}_{2},\;O_{2},\;\mathrm{biomass}\right\},$$

where

(3d) $$\rho_{j}=\sum_{i=1}^{n}\lambda_{i}^{\left(1\right)}\delta_{ij},$$

(3e) $$\delta_{ij}=\left\{\begin{array}{cc} 1 & \text{if}\;e_{j}^{i}>0,\\ 0 & \text{otherwise}, \end{array}\right.$$

(3f) $$\sigma_{u}=\sum_{i=1}^{n}\lambda_{i}^{\left(1\right)}\varepsilon_{iu},$$

(3g) $$\varepsilon_{iu}=\left\{\begin{array}{cc} 1 & \text{if}\;Y_{u}^{i}\geq Y_{\min,u},\\ 0 & \text{otherwise}, \end{array}\right.$$

and

(3h) $$\Delta_{r}G_{j}=\sum_{k=1}^{m}\Delta_{f}G_{k}^{\prime }S_{kj},$$

(3i) $$\Delta_{f}G_{k}^{\prime }=\Delta_{f}G_{k}^{\prime 0}+RT\ln\left(c_{k}/c_{0}\right),\;c_{0}=1M,$$

(3j) $$\ln\left(c_{k}^{\min}/c_{0}\right)\leq \ln\left(c_{k}/c_{0}\right)\leq \ln\left(c_{k}^{\max}/c_{0}\right).$$

Here σ_*u*_ counts the number of EFMs that have a certain minimal yield of metabolite *u*, cf. Eqn (3f), and ɛ_*iu*_ indicates if EFM *i* has the required yield for metabolite *u*, cf. Eqn (3g). The constraint (Eqn 3c) ensures that at least one EFM has the required yield. All other subequations are also found in the original MILP (Eqn (1a), (1b), (1c), (1d), (1e), (1f), (1g)).

### Expression data further reduces the number of relevant LTCSs

We further analyzed the remaining 12 LTCSs, using expression data. Six LTCSs, ℒ_8_,ℒ_12_,ℒ_15_,ℒ_16_,ℒ_21_ and ℒ_38_, had an active fumarate reductase (FrdABCD), but had an inactive succinate dehydrogenase (SdhCDAB) in all their EFMs. This is in contrast to experimental findings since, under aerobic conditions, sdhCDAB is optimally expressed 32 while the frdABCD operon is repressed 33. Thus only six LTCSs, ℒ_1_,ℒ_3_,ℒ_5_,ℒ_6_,ℒ_7_ and ℒ_18_, were found to be consistent with the data.

**Figure 6:**
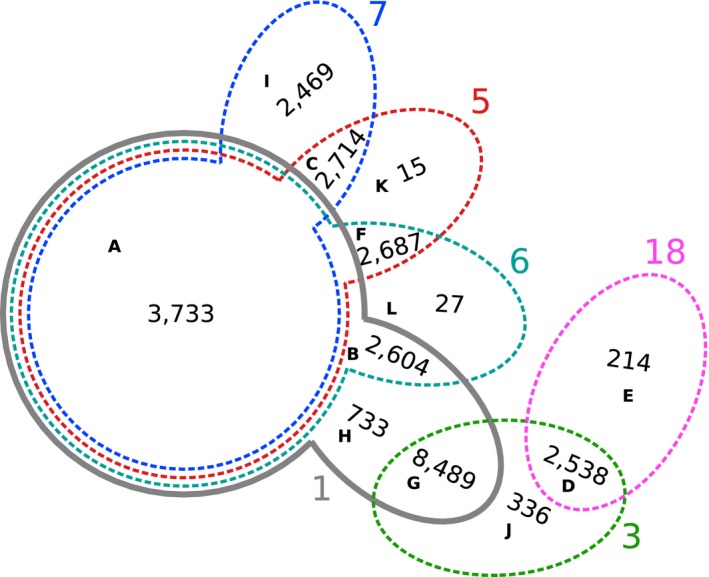
Venn diagram for LTCSs ℒ_1_, ℒ_3_, ℒ_5_, ℒ_6_, ℒ_7_, and ℒ_18_. Each LTCS is denoted by its set index and printed in the same color as in Fig. 7. The letters A to L denote different segments in the diagram, along with the number of TF EFMs in these segments. Only ℒ_1_ (full line) was found to be consistent with yield, expression, and flux data. All other LTCSs (dashed lines) were eliminated, which turned out to be consistent with independent ^13^C flux data.

**Figure 7:**
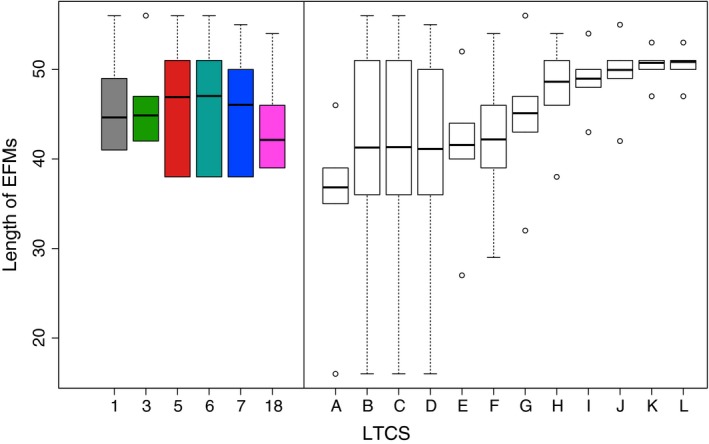
Boxplots showing the distribution of the cardinalities of the TF EFMs in the LTCSs shown in Fig. 6 as function of the set index (left panel) and the segment label of the Venn diagram in Fig. 6 (right panel).

The Venn diagram in Fig. 6 singled out ℒ_3_ and ℒ_18_ due to their lack of overlap with the other LTCSs (see the next section for a mechanistic characterization of these sets). In contrast to ℒ_3_ and ℒ_18_, the four remaining LTCSs share some core functionality, represented by a large fraction of common EFMs (segment A). We investigated if these EFMs were characterized by their supports and observed that on average shared EFMs (contained in several LTCSs) were shorter than EFMs unique to an LTCS (see Fig. 7). Functionally, EFMs in segment A do not invoke the pentose phosphate pathway and do not produce biomass. However, some produce maintenance energy (see Table 1). In all other segments, biomass production is feasible. For a complete listing see Table 2.

**Table 1:** Relative frequency (in %) of EFMs in segments A–L of the Venn plot in Fig. 6 supported by certain reactions. Negative numbers indicate that reactions are used in the backward direction. Reactions are either specified explicitly or represented by their SMBL id. The SBML file of the metabolic model is available at https://github.com/mpgerstl/ltcsCalculator and the comprehensive list for all reactions is available in Table 2. Ery4*P*, erythrose 4‐phosphate; Fru6*P*, fructose 6‐phosphate; Gra3*P*, glyceraldehyde 3‐phosphate; Glc6*P*, glucose 6‐phosphate; R_ATPM, non‐growth‐associated ATP maintenance reaction; Xyl5*P*, xylulose 5‐phosphate

Gene or function	Reaction	A	B	C	D	E	F	G	H	I	J	K	L
Biomass production	R_BIOMASS	0	100	100	23	3.7	100	17	64	100	95	100	100
Maintenance energy	R_ATPM	18	2	3	11	3.7	3	15	6	3	2	0	0
*pgi*	Glc6*P* ⇌ Fru6*P*	100	100	100	−100	−100.0	100	0	100	100	−100	100	100
*tktA*,* tktB*	Ery4*P* + Xyl5*P* ⇌ Fru6*P* + Gra3*P*	0	0	−100	100	100.0	−100	100	100	−100	100	−100	−100

**Table 2:** Relative frequency (%) of reactions supporting the EFMs in the segments (A–L) of the Venn plot in Fig. 6. Negative numbers indicate that reactions are used in the backward direction. Reactions are grouped in biologically defined subsystems

Reaction	A	B	C	D	E	F	G	H	I	J	K	L
Anaplerotic reactions												
R_ME2	44	37	38	16	7.5	37	27	21	43	6	40	4
R_ME1	34	30	30	16	7.5	30	27	26	35	10	20	26
R_MALS	50	57	55	63	60.7	56	54	63	54	73	0	37
R_ICL	50	57	55	63	60.7	56	54	63	54	73	0	37
R_PPC	81	88	88	46	13.1	88	85	90	87	39	100	100
R_PPCK	17	3	4	23	23.4	4	15	7	4	1	0	0
Biomass												
R_BIOMASS	0	100	100	23	3.7	100	17	64	100	95	100	100
Citric acid cycle												
R_AKGDH	45	48	49	19	15.0	48	32	18	49	12	87	59
R_CS	97	100	100	90	77.6	100	97	99	100	100	100	100
R_ACONTb	97	100	100	90	77.6	100	97	99	100	100	100	100
R_ACONTa	97	100	100	90	77.6	100	97	99	100	100	100	100
R_MDH	32	37	36	57	68.2	37	37	34	30	77	33	56
R_ICDHyr	57	100	100	56	33.6	100	64	85	100	95	100	100
R_FUM	85	67	67	63	60.7	67	53	26	82	67	80	74
R_SUCOAS	−45	−48	−49	−19	−15.0	−48	−32	−18	−49	−12	−87	−59
Exchange												
R_EX_for_e	47	62	64	48	42.5	63	50	64	64	54	100	96
R_EX_pi_e	0	−100	−100	−23	−3.7	−100	−17	−64	−100	−95	−100	−100
R_EX_h_e	84	100	100	85	69.6	100	92	100	100	100	100	100
R_EX_gln_L_e	0	0	0	0	0.0	0	0	0	0	0	0	0
R_EX_glyc_e	0	0	0	0	0.0	0	0	0	0	0	0	0
R_EX_nh4_e	−8	−100	−100	−35	−11.2	−100	−29	−76	−100	−95	−100	−100
R_EX_bm	0	100	100	23	3.7	100	17	64	100	95	100	100
R_EX_mal_L_e	0	0	0	0	0.0	0	0	0	0	0	0	0
R_EX_glu_L_e	8	7	6	13	7.5	6	14	20	6	6	0	7
R_EX_pyr_e	14	20	19	7	3.7	20	16	18	15	12	13	15
R_EX_fru_e	0	0	0	0	0.0	0	0	0	0	0	0	0
R_EX_o2_e	−100	−100	−100	−100	−100.0	−100	−100	−100	−100	−100	−100	−100
R_EX_fum_e	0	0	0	0	0.0	0	0	0	0	0	0	0
R_EX_h2o_e	99	100	100	100	100.0	100	100	100	100	100	100	100
R_EX_lac_D_e	13	20	20	6	2.8	20	16	18	16	12	20	11
R_EX_succ_e	16	35	35	19	15.0	35	34	56	28	19	13	19
R_EX_akg_e	7	29	32	9	7.5	31	14	14	33	6	93	93
R_EX_co2_e	94	65	61	100	100.0	62	100	72	62	100	7	4
R_EX_glc_e	−100	−100	−100	−100	−100.0	−100	−100	−100	−100	−100	−100	−100
R_EX_ac_e	16	20	19	9	7.5	20	17	20	15	12	7	15
R_EX_acald_e	0	0	0	0	0.0	0	0	0	0	0	0	0
R_EX_etoh_e	0	0	0	0	0.0	0	0	0	0	0	0	0
Glutamate metabolism												
R_GLNS	41	100	100	53	18.7	100	55	85	100	97	100	100
R_GLUN	18	2	3	11	3.7	3	15	6	3	2	0	0
R_GLUSy	25	100	100	44	15.0	100	42	81	100	96	100	100
R_GLUDy	18	2	3	11	3.7	3	15	6	3	2	0	0
Glycerolipid metabolism												
R_G3PD2	−61	−31	−29	−64	−63.1	−30	−64	−53	−29	−66	0	−15
R_GLYK	0	0	0	0	0.0	0	0	0	0	0	0	0
R_G3PD5	61	31	29	64	63.1	30	64	53	29	66	0	15
Glycolysis/gluconeogenesis												
R_PYK	26	30	29	16	0.9	29	19	22	37	4	7	4
R_GAPD	100	100	100	100	91.6	100	100	100	100	100	100	100
R_PDH	52	47	47	48	42.5	47	49	36	45	46	20	19
R_TPI	100	100	100	0	−100.0	100	100	100	100	100	100	100
R_PGK	−100	−100	−100	−100	−91.6	−100	−100	−100	−100	−100	−100	−100
R_PFK	100	100	100	11	3.7	100	100	100	100	100	100	100
R_PGM	−100	−100	−100	−100	−91.6	−100	−100	−100	−100	−100	−100	−100
R_PGI	100	100	100	−100	−100.0	100	0	100	100	−100	100	100
R_ENO	100	100	100	100	91.6	100	100	100	100	100	100	100
R_FBP	18	2	3	11	100.0	3	15	6	3	2	0	0
R_FBA	100	100	100	0	−100.0	100	100	100	100	100	100	100
R_PPS	23	13	13	53	77.6	13	21	15	8	3	27	4
Inorganic ion transport and metabolism												
R_NH4t	8	100	100	35	11.2	100	29	76	100	95	100	100
R_PIt2r	0	100	100	23	3.7	100	17	64	100	95	100	100
Oxidative phosphorylation												
R_NADTRHD	34	19	17	45	46.7	18	45	7	17	43	0	15
R_CYTBD	100	100	100	100	100.0	100	100	100	100	100	100	100
R_ADK1	23	13	13	53	77.6	13	21	15	8	3	27	4
R_ATPM	18	2	3	11	3.7	3	15	6	3	2	0	0
R_NADH16	59	84	85	63	60.3	85	63	54	84	60	100	100
R_ATPS4r	22	35	38	34	26.2	37	27	24	38	27	100	78
R_SUCDi	85	67	67	63	60.7	67	53	26	82	67	80	74
R_THD2	52	41	40	45	44.9	40	46	57	41	49	0	11
R_FRD7	0	0	0	0	0.0	0	0	0	0	0	0	0
Pentose phosphate pathway												
R_TKT2	0	0	−100	100	100.0	−100	100	100	−100	100	−100	−100
R_GND	0	100	100	100	100.0	100	100	100	6	100	100	100
R_TKT1	0	100	0	100	100.0	100	100	100	−100	100	100	100
R_PGL	0	100	100	100	100.0	100	100	100	6	100	100	100
R_RPI	0	−100	−100	−100	−100.0	−100	−100	−100	−100	−100	−100	−100
R_RPE	0	100	−100	100	100.0	0	100	100	−100	100	−100	100
R_G6PDH2r	0	100	100	100	100.0	100	100	100	6	100	100	100
R_TALA	0	100	0	100	100.0	100	100	100	−100	100	100	100
Pyruvate metabolism												
R_ACKr	−16	−20	−19	−9	−7.5	−20	−17	−20	−15	−12	−7	−15
R_ACALD	0	0	0	0	0.0	0	0	0	0	0	0	0
R_ALCD2x	0	0	0	0	0.0	0	0	0	0	0	0	0
R_PTAr	16	20	19	9	7.5	20	17	20	15	12	7	15
R_PFL	47	62	64	48	42.5	63	50	64	64	54	100	96
R_LDH_D	−13	−20	−20	−6	−2.8	−20	−16	−18	−16	−12	−20	−11
Transport/extracellular												
R_GLUt2r	−8	−7	−6	−13	−7.5	−6	−14	−20	−6	−6	0	−7
R_FUMt2_2	0	0	0	0	0.0	0	0	0	0	0	0	0
R_ETOHt2r	0	0	0	0	0.0	0	0	0	0	0	0	0
R_FRUpts2	0	0	0	0	0.0	0	0	0	0	0	0	0
R_GLYCt	0	0	0	0	0.0	0	0	0	0	0	0	0
R_FORt2	22	22	22	26	28.5	22	27	12	22	26	0	7
R_SUCCt3	36	49	49	40	37.9	49	52	61	43	41	13	26
R_GLNabc	0	0	0	0	0.0	0	0	0	0	0	0	0
R_CO2t	−94	−65	−61	−100	−100.0	−62	−100	−72	−62	−100	−7	−4
R_H2Ot	−99	−100	−100	−100	−100.0	−100	−100	−100	−100	−100	−100	−100
R_PYRt2r	−14	−20	−19	−7	−3.7	−20	−16	−18	−15	−12	−13	−15
R_GLCpts	100	100	100	100	100.0	100	100	100	100	100	100	100
R_ACt2r	−16	−20	−19	−9	−7.5	−20	−17	−20	−15	−12	−7	−15
R_FORti	59	71	72	61	58.4	72	64	66	72	67	100	96
R_AKGt2r	−7	−29	−32	−9	−7.5	−31	−14	−14	−33	−6	−93	−93
R_D_LACt2	−13	−20	−20	−6	−2.8	−20	−16	−18	−16	−12	−20	−11
R_SUCCt2_2	22	22	22	26	28.5	22	27	12	22	26	0	7
R_ACALDt	0	0	0	0	0.0	0	0	0	0	0	0	0
R_MALt2_2	0	0	0	0	0.0	0	0	0	0	0	0	0
R_O2t	100	100	100	100	100.0	100	100	100	100	100	100	100

### Flux data pinpoint a single relevant LTCS

The metabolic capabilities of *E. coli* when grown aerobically on minimal medium under glucose limited conditions are fully described by the six LTCSs in Fig. 6. To further narrow down the number of LTCSs, we analyzed the different segments in Fig. 6, using flux data.

All EFMs in the segments D, E and J (see ℒ_3_ and ℒ_18_ in Fig. 6) were characterized by a reverse flux across glucose‐6‐phosphate isomerase (Pgi), directed towards glucose 6‐phosphate (see Table 1). Under the standard growth conditions investigated here, Pgi is forward active 34. Thus we were able to eliminate $L_{3}$ and ℒ_18_ from the set of relevant LTCSs. (Note that segment G, which is the largest subset of ℒ_3_, was not removed since EFMs in G have zero flux across Pgi.).

We further investigated if the remaining segments (A, B, C, F, G, H, I, K and L) could be distinguished by the directions of reversible reactions. In particular, we analyzed the flux across transketolase (TktA, TktB) and found that all EFMs in the segments A and B carried no flux, whereas all EFMs in the segments C, F, I, K and L had a reverse flux and all EFMs in G and H had a forward flux. Under the standard growth conditions investigated here, transketolase is forward active 34. Thus we concluded that ℒ_1_ is the only biologically relevant LTCS.

In ℒ_1_ (as in every other LTCS) all reversible reactions have a fixed direction (due to the no‐cancelation rule). The predicted directions were fully consistent with independent ^13^C flux data 35.

To summarize, we found that from 40 LTCSs only ℒ_1_ was consistent with all data. ℒ_1_ contains 15 559 TF EFMs, which represent only 9% of all EFMs. More specifically, 4486 EFMs produce biomass, 2024 produce maintenance energy, and 54 produce both. In fact, ℒ_1_ is composed of several segments: all EFMs in segment A do not invoke the pentose phosphate pathway and do not produce biomass, but are responsible for the production of maintenance energy; all EFMs in segment B produce biomass without invoking TktA or TktB; all EFMs in segment G do not carry flux across Pgi, but are able to produce biomass and/or maintenance energy. Finally, EFMs in segment H are not characterized by a single common property.

### General remarks on LTCSs

Using *E. coli* as an example, we outlined a procedure that narrowed down the feasible solution space and eventually identified a single LTCS. The success of such an analysis is dependent on the quality of the measured metabolome. However, the general concept of LTCSs is not affected by the metabolome's quality. In fact, we can find LTCSs even if the metabolome is unknown. As soon as a network contains at least two EFMs that are supported by a reversible reaction carrying fluxes in opposite directions, different LTCSs exist. Moreover, the cardinalities of these LTCSs are always smaller than the total number of EFMs in a system (see for instance Fig. 1, where $4\;=\;n^{\text{tot}}\;>$ |ℒ_1_| = |ℒ_2_| = 3). Thus even in the absence of a measured metabolome it is useful to look at LTCSs, as only then is a thermodynamically consistent understanding guaranteed. Although an LTCS is less complex than the complete set of EFMs, one now has to analyze multiple LTCSs. In general the number of LTCSs scales combinatorially with the number of reversible reactions in a network. Practically, that is why an accurately measured metabolome is essential.

## Discussion

Every intercellular flux distribution is TF and can be decomposed into TF EFMs. However, the reverse is not necessarily true. That is, the conformal superposition of any two TF EFMs is itself not necessarily TF.

Here we developed a method that identifies the largest sets of TF EFMs that are thermodynamically consistent (LTCSs). Within an LTCS every nonnegative linear combination of its elements results in a TF flux distribution. A necessary condition for an LTCS is that all reactions supporting the EFMs of an LTCS operate in the same direction. This is known as the no‐cancelation rule 2, 3.

**Figure 8:**
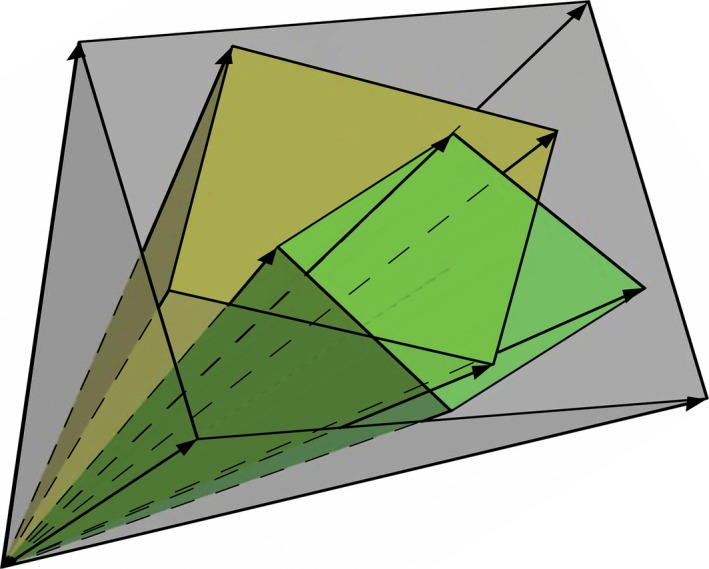
Graphical representation of the segmented steady‐state flux cone (grey) containing two partially overlapping subcones spanned by LTCSs.

Geometrically, an LTCS spans a TF subcone of the flux cone (see Fig. 8). In fact, thermodynamic constraints segment the flux cone into LTCSs. Although subcones corresponding to LTCSs may overlap (see Fig. 6), each LTCS has unique metabolic capabilities.

We found that shorter TF EFMs are more likely to be elements in multiple LTCSs than longer TF EFMs. More specifically, we found that segment A in Fig. 6 contains only EFMs producing maintenance energy, but no EFM producing biomass. This can be understood considering the number of reactions involved. Whereas biomass requires the production of many precursors which involves long pathways, maintenance energy requires only the production of adenosine triphosphate (ATP), which can be achieved by short routes. Since every reaction has to comply with the second law of thermodynamics, the likelihood of thermodynamical feasibility decreases with increasing number of contributing reactions.

Every metabolic phenotype can be described by an LTCS. Conversely, if an EFM is not part of an LTCS, it is biologically irrelevant, since it does not contribute to a thermodynamically consistent decomposition of a TF flux distribution. In general, decompositions into EFMs are not unique, and several methods have been proposed 36, 37, 38, 39, 40. However, none of these methods takes thermodynamics into account, which may lead to inconsistent decompositions. Therefore, it is even more important to identify those LTCSs that consistently describe a phenotype.

We outlined a systematic procedure to identify biologically relevant LTCSs. Based on the integration of additional (omics) data, we successively narrowed down the number of LTCSs. In fact, most LTCSs were found to be inconsistent with commonly available growth parameters. Further consideration of expression and flux data eventually identified a single LTCS that characterizes the phenotype. The additional information could have been used to adapt the network first. Doing so would have reduced the number of LTCSs from 40 to four (ℒ_1_, ℒ_2_, ℒ_31_, and ℒ_36_), and a comparison with growth parameters would have identified ℒ_1_, the same LTCS as before. However, for less studied organisms detailed data may not be available. In this case, an analysis of phenotypical properties like in Tables 1 and 2 will identify the most valuable piece of information to narrow down the number of LTCSs.

Our method is able to compute LTCSs if all TF EFMs are known, and we showed that the set of TF EFMs characterizing a phenotype is smaller than the set of all TF EFMs. However, currently our method does not allow computation of LTCSs directly. It would be desirable to enumerate only the biologically relevant EFMs, which would facilitate an unbiased analysis of metabolic systems even on a genome‐scale level. Recent progress enabled the selective calculation of subsets of EFMs 21 and the identification of relevant regulated EFMs 41, 42. Combining these ideas with our current approach may lead to promising lines of future research.

## Methods

We used the software package tefma 24 together with published metabolite concentration data 43 to calculate all TF EFMs in a core metabolic model of *E. coli* 25 growing on a glucose limited minimal medium. For all unmeasured metabolites in this model we used conservative default concentration ranges between $c_{k}^{\min}\;=\;10^{-7}\;m$ and $c_{k}^{\max}\;=\;1\;m$. Δ_f_ *G* ^0^ values were obtained from the online version of equilibrator 44. For two metabolites (ubiquinol‐8 and biomass) no Δ_f_ *G* values were available. Reactions to which those metabolites contributed were not checked for thermodynamic feasibility to avoid false conclusions 25.

The set of Eqns (1a), (1b), (1c), (1d), (1e), (1f), (1g) and (2) were solved with the IBM ilog cplex Optimization Studio, version 12.5. A Perl‐script that sets up the systems equations and invokes the cplex lp solver, and the metabolic model and all data used in this study are available at https://github.com/mpgerstl/ltcsCalculator. Note that cplex is a commercial software product although free academic licenses are available.

## Author contributions

J.Z. conceived the study; all authors contributed to the development of the theory; J.Z. and M.P.G. designed the study; M.P.G. developed the software and performed the analyses; all authors wrote, read and approved the final manuscript.
